# Changes in Skin Barrier Function after Repeated Exposition to Phospholipid-Based Surfactants and Sodium Dodecyl Sulfate In Vivo and Corneocyte Surface Analysis by Atomic Force Microscopy

**DOI:** 10.3390/pharmaceutics13040436

**Published:** 2021-03-24

**Authors:** Claudia Vater, Alexandra Apanovic, Christoph Riethmüller, Brigitte Litschauer, Michael Wolzt, Claudia Valenta, Victoria Klang

**Affiliations:** 1 Department of Pharmaceutical Technology and Biopharmaceutics, Faculty of Life Sciences, University of Vienna, 1090 Vienna, Austria; claudia.vater@univie.ac.at (C.V.); a01201528@unet.univie.ac.at (A.A.); claudia.valenta@univie.ac.at (C.V.); 2 Research Platform “Characterisation of Drug Delivery Systems on Skin and Investigation of Involved Mechanisms”, University of Vienna, 1090 Vienna, Austria; 3 Serend-ip GmbH, Centre for Nanotechnology, 48149 Münster, Germany; info@serend-ip.de; 4 Department of Clinical Pharmacology, Medical University of Vienna, 1090 Vienna, Austria; brigitte.litschauer@meduniwien.ac.at (B.L.); michael.wolzt@meduniwien.ac.at (M.W.)

**Keywords:** surfactants, phospholipids, lecithins, sodium dodecyl sulfate, atomic force microscopy, corneocytes, transepidermal water loss, natural moisturizing factor, skin hydration, dermal texture index

## Abstract

(1) Background: The aim of the study was to evaluate the effect of pure lecithins in comparison to a conventional surfactant on skin in vivo. (2) Methods: Physiological skin parameters were evaluated at the beginning and the end of the study (day 1 and day 4) (*n* = 8, healthy forearm skin) with an Aquaflux^®^, skin-pH-Meter, Corneometer^®^ and an Epsilon^®^ sensor. Confocal Raman spectroscopy was employed to monitor natural moisturizing factor, urea and water content of the participants’ skin. Tape strips of treated skin sites were taken and the collected corneocytes were subjected to atomic force microscopy. Circular nano objects were counted, and dermal texture indices were determined. (3) Results: Transepidermal water loss was increased, and skin hydration was decreased after treatment with SDS and LPC80. Natural moisturizing factor and urea concentrations within the outermost 10 µm of the stratum corneum were lower than after treatment with S75 or water. Dermal texture indices of skin treated with SDS were higher than skin treated with water (control). (4) Conclusions: Results suggest very good (S75) or good (LPC80) skin-tolerability of lecithin-based surfactants in comparison to SDS and encourage further investigation.

## 1. Introduction

The human skin is a complex organ that comprises different layers with specific physiology and functions. The outermost layer, the epidermis, represents the main barrier against the surrounding environment—it prevents the ingress of chemicals or microorganisms and at the same time prevents excessive loss of water [1,2,3,4]. The outermost layer of the epidermis, termed the stratum corneum (SC), plays a key role in this context. It is composed of 10 to 25 stacked layers of flattened, elongated, keratinized dead cells, the corneocytes, which are embedded in a complex lipid matrix [1,3]. Corneocytes emerge from the main cell type of the living epidermis, the keratinocytes, which are formed in the basal layer of the epidermis and then constantly migrate towards the skin surface. During this process, differentiation occurs and the ultimately emerging corneocytes are subjected to desquamation [3]. The intercellular lipid matrix, which mainly consists of ceramides, free fatty acids and cholesterol, is essential in maintaining the barrier function. Its specific composition and lipid organisation is pivotal and has been subject to intensive scientific examination [1,2,3,5,6,7].

The functioning of the SC barrier, which can be imaged as a “brick and mortar” type wall [2,3,8], depends on the intactness of its structural composition. When the SC is confronted with chemicals that possess surface-active activities, it can be compromised. Many of the daily products that we apply onto our skin for cosmetic purposes, e.g., cosmetic creams or cleaning products such as soaps, washing detergents, shower gels or shampoos, contain surfactants—either as essential structural compound or as active principle in the removal of dirt [9].

In general, surfactants are key excipients in pharmaceutical and cosmetic formulation development [9,10,11]. Due to increased consumer interest and environmental aspects, there is a constant tendency towards the use of surfactants from renewable raw materials. Global interest in sustainable surface-active agents is growing, especially in regard to sensitive routes of application such as mucosae or skin. In contrast, interest in classic anionic sufactants such as sodium dodecyl sulfate (SDS) has decreased, although certain derivatives such as sodium lauryl ether sulfate (SLES) are still omnipresent in marketed products. Classic SDS is a versatile anionic surfactant with a large polar sulfate head group; its (hydrophilic-lipophilic balance) HLB value can be calculated as being around 40. After years of intensive use in pharmaceutical technology, there have been increasing numbers of scientific investigations reporting negative effects on skin physiology. Treatment of the skin with SDS has been shown to increase transepidermal water loss (TEWL) [12,13,14] and to affect keratinocyte differentiation and desquamation [15,16]. SDS not only affects SC integrity, but also acts on deeper skin layers, where it is able to trigger skin irritations and allergies [11,17,18].

Thus, attention has shifted towards exploring potential alternatives. Among the current surfactants of interest, we have been focusing on the use of amphoteric phospholipid-based surfactants. Naturally occuring lecithin mixtures have been used as additives in the food industry for decades and are gaining popularity in the pharmaceutical and cosmetic sector as a result of increased environmental awareness [19,20]. The term lecithin, originally referring to phosphatidylcholine itself, is today used to describe a mixture of various phospholipids with adherent glycerol and fatty acids. The commonly used lecithin mixtures exhibit HLB values of 7 to 9 [21]. In this study, different lecithin mixtures were used. The main compounds of importance, phosphatidylcholine and monoacyl-phosphatidylcholine, which is produced from the former through hydrolysis, are shown in Supplementary Figure S1.

The skin-friendly nature of lecithin mixtures in formulation development is well established from a practical viewpoint [22,23,24,25]. However, many data were obtained ex vivo while using complex formulations or in vitro in cell culture studies [26,27,28]. There is little data on direct evaluation of pure excipients in vivo on human skin on a molecular level.

Thus, the aim of the present study was to investigate the effect of two different lecithin-based surfactants and SDS on physiological skin parameters, SC compounds and corneocyte morphology after repeated exposure in vivo on human forearm skin. 

Classic biophysical methods were used to measure TEWL (closed-chamber method), SC hydration (two permittivity-based methods) and skin surface pH. Confocal Raman spectroscopy (CRS) was used to investigate the surfactants’ effects on relative natural moisturising factor (NMF) levels in general and urea levels in particular; the overall water content was analyzed as well. 

To complement these well-established techniques [29,30,31,32,33], changes in corneocyte surface morphology were evaluated by atomic force microscopy (AFM). This technique has recently been adapted for skin analysis and has been successfully used in several studies [34,35,36,37]. The underlying principle is the determination of the dermal texture index (DTI), which is based on the visual observation and counting of pathological protrusions on the corneocytes’ surface [36]. These so-called circular nano objects (CNOs) are often found in patients with chronic pathological skin conditions such as atopic dermatitis, but also after treatment with possible irritants [36,38]. 

The study design involved an initial assessment of all biophysical parameters and spectroscopic data on eight healthy volunteers. Treatment with the three different surfactant dispersions (1% *w*/*w* of SDS, Lipoid^®^ S75 or Lipoid^®^ S LPC80) was performed on four subsequent days, with two treatments of 30 min under occluded conditions each day. Purified water was applied as a control in the same manner. After the final treament on day four, the parameters of interest were determined again. In addition, corneocyte samples were taken from the skin surface of the treatment and control areas via tape stripping in order to perform AFM analysis. Five consecutive tapes were removed from each site and subjected to AFM analysis to evaluate corneocyte surface morphology against the control.

## 2. Materials and Methods

### 2.1. Materials

Soybean lecithin Lipoid^®^ S75 (soybean derived phospholipids with phosphatidylcholine content of 70%) and Lipoid^®^ S LPC80 (soybean derived phospholipids with 80% monoacyl-phosphatidylcholine content) were kindly provided by Lipoid GmbH (Ludwigshafen, Germany). SDS was purchased from Sigma Aldrich (Sigma-Aldrich, St. Louis, MO, USA). Standard Corneofix^®^ adhesive films with a square area of 4.0 cm^2^ were obtained from Courage and Khazaka GmbH (Cologne, Germany). Finn Chambers on Scanpor (12 mm in diameter) were purchased from Smart Practice, Barsbüttel, Germany.

### 2.2. Study Design

The study was approved by the Ethics Committee of the Medical University of Vienna (approval number: 1213/2020) in accordance with the guidelines of the Declaration of Helsinki. Effect of the surfactants on physiological skin parameters was evaluated with the cooperation of eight healthy volunteers of both sexes (four female, four male, aged between 24 and 32 years). Volunteers gave their informed written consent to participate. Individuals with chronic skin conditions, skin lesions, tattoos or scars on their forearms were excluded from participation. Additionally, participants were told not to use any skin care products on their forearms for the duration of the study and to avoid excessive sporting activities before measurements. 

The study design is visualized in Supplementary Figure S2. Participants were randomly classified as S001-S008. The non-dominant volar forearms were exposed to 50 µL of aqueous solution of 1% sodium dodecyl sulfate (SDS), 1% liposomal dispersions of lecithins Lipoid^®^ S75 and Lipoid^®^ S LPC80 and distilled water, in a repeated irritation test (twice a day for 30 min) over a period of four days. TEWL, skin hydration, skin permittivity, skin pH and NMF, urea and water levels were measured at baseline and after four days. Exposure to the irritants and distilled water as a control was performed under occlusion with large Finn Chambers on Scanpor (12 mm in diameter; Smart Practice, Barsbüttel, Germany). Volunteers were allowed to acclimatize for 15 min before measurements at 22.6 ± 1.3 °C ambient temperature and 37.3 ± 6.9% relative humidity. On day 4, after the last skin parameter measurement, five consecutive tape strips were taken from each test site and stored at room temperature until AFM analysis. 

### 2.3. Effect of Surfactants on Skin Parameters

#### 2.3.1. Transepidermal Water Loss (TEWL)

TEWL measurements were performed with the condenser-chamber device AquaFlux^®^ AF200 (Biox Ltd., London, UK). With its closed-chamber principle the AquaFlux^®^ creates a microclimate within the measuring compartment, rendering the measurements unaffected by external air turbulences. The device measures water evaporating from the skin in g/m^2^/h. Increased water loss indicates skin barrier damage [39,40]. Measurements were performed in triplicate.

#### 2.3.2. Skin Surface pH

For skin pH measurements, the Skin-pH-Meter PH 905 (Courage + Khazaka electronic GmbH, Cologne, Germany), mounted on a combination Derma unit SSC3 (Courage + Khazaka electronic GmbH, Cologne, Germany), was used. Following EEMCO guidelines, the electrode was calibrated regularly and volunteers were given time to acclimatize [41]. Measurements were performed in triplicate.

#### 2.3.3. Skin Hydration 

Skin hydration measurements were conducted using a Corneometer^®^ CM 825 (Courage + Khazaka electronic GmbH, Cologne, Germany) mounted on a combination Derma unit SSC3 (Courage + Khazaka electronic GmbH, Cologne, Germany). The device measures the electrical capacitance of the skin surface, thus indirectly measuring skin hydration, as the electrical capacitance depends on the water content [42]. Measurements were performed in triplicate.

#### 2.3.4. Capacitive Contact Imaging (Skin Permittivity)

The fingerprint sensor Epsilon^®^ (Biox Ltd., London, UK) was used to visually assess skin hydration by capacitive contact imaging. The device consists of 76,800 capacitive sensors (12.8 × 15 mm area, 50 µm image resolution) and has a measurement depth of approximately 20 µm. The measured parameter is the dielectric permittivity ε, displayed in real-time high-resolution images. Areas with no electric permittivity (ε = 0), such as furrows, are illustrated in black, while bright areas indicate high permittivity, and therefore, high skin hydration [43]. Measurements were performed in triplicate.

#### 2.3.5. Confocal Raman Spectroscopy (CRS)

CRS experiments were performed using a confocal Raman microspectrometer (gen2 Skin Composition Analyzer, River Diagnostics, Rotterdam, The Netherlands) with two incorporated lasers, operating at different wavelengths (785 nm for analysis of the skin fingerprint region (400–1800 cm^−^^1^) and 671 nm for analysis of the high wave number region (2500–4000 cm^−^^1^)). The device has been specifically developed for the in vivo analysis of human skin, and is able to monitor treatment-induced changes in skin parameters such as NMF, urea or water content. CRS measurements were performed at baseline on day one and after the last 30 min of exposure to the three test substances and the control on day four. Measurements of the fingerprint region were performed in 2 µm depth increments up to a depth of 30 µm at a signal collection time of 5 s per spectrum. Measurements in the high wave number region were conducted in 2 µm depth increments up to a depth of 40 µm at an exposure time of 2 s per spectrum. Data were collected up to 40 µm skin depth. For final evaluation, only the first 10 µm of the SC were considered relevant due to the higher accuracy of data collection in these depths. Likewise, changes in SC parameters are of highest interest in this region. At least *n* = 5 measurements were performed in the fingerprint region and at least *n* = 3 measurements were performed in the high wavenumber region to ensure acceptable measurement duration and avoid occlusion effects due to prolonged measurement sessions. Experiments were executed on the volunteers’ volar forearms on random positions within the area of interest on day one. On day four, the same number of measurements were performed for each test substance’s region of interest. For further analysis, the obtained spectra of each participant were averaged and evaluated using SkinTools^®^ software version 2.0 (River Diagnostics, The Netherlands). For the calculation of the relative concentrations of NMF and urea, we used a non-restricted multiple least-square fitting algorithm with the epidermis’ dominant protein keratin as an internal standard, as the concentration of keratin is assumed to stay relatively constant throughout the SC [44]. Water profiles were generated by calculating the water content from the water to protein ratio [44]. 

### 2.4. Calculation of Skin Parameter Changes

The effect of repeated treatment with the different surfactants on skin parameters, such as TEWL, skin surface pH, skin hydration, urea and NMF content, should be evaluated and compared. To this end, the following Equation (1) was employed:

(1) $$Skin\;parameter\;change\;\left[\%\right]=\left(\frac{T}{C}-1\right)\times 100\;$$

in which *T* refers to the mean values of the treated sites after the last exposure to the different test substances, while *C* refers to the mean values of the untreated forearm at the beginning of the study.

### 2.5. Effect of Surfactants on Corneocyte Surface Morphology

#### 2.5.1. Sampling of the SC by Tape Stripping

SC samples for AFM analysis were collected by tape stripping. The Corneofix^®^ adhesive tape strips (Courage and Khazaka GmbH, Germany) were placed on the designated areas. The tape stripping process was performed on a scale to ensure a constant pressure of 49 N (5 kg). After applying pressure for three seconds, the tape was removed in a single rapid movement. Five tape strips of every region of interest per volunteer were collected in this fashion, placed in a closed vial and stored at room temperature until analysis.

#### 2.5.2. Analysis of Corneocyte Surface by Atomic Force Microscopy (AFM)

The corneocytes adherent to the tape strips were analyzed by AFM as described [36,45]. In brief, each third consecutive tape strip was subjected to AFM analysis carried out with a multimode atomic force microscope equipped with a Nanoscope III controller and software version 5.30 sr3 (Digital Instruments, Santa Barbara, California). Silicon-nitride tips on V-shaped gold-coated cantilevers were used (0.01 N/m, MLCT, VEECO, Mannheim, Germany). Imaging was performed in air at ambient temperature with forces less than 1 nN at one to three scan lines per second (1–3 Hz) with a resolution of 512 × 512 pixels. For nano-object analysis, sub-cellular scan areas of 20 × 20 μm^2^ were recorded. Topographical data of the cell surfaces were analyzed using the nAnosticTM-method applying custom-built, proprietary algorithms (Serend-ip GmbH, Muenster, Germany). Nanostructures protruding from the mean surface level are referred to as CNOs. CNOs of size < 500 nm were counted, the average object count of 10 areas is referred to as DTI [36,37]. 

### 2.6. Statistical Analysis

Results are expressed as mean values ± standard deviation (SD). Data were analyzed using the JASP 0.9.0.1 software using the Shapiro-Wilk test as test of normality and one-way ANOVA + post-hoc Tukey test or the Student’s t-test with *p* < 0.05 as minimum level of significance.

## 3. Results

### 3.1. Effect of Surfactants on Skin In Vivo

The study was implemented as planned and all volunteers completed the study. Two of the participants (S005 and S006) showed a very strong or strong reaction to SDS, which occurred on the last day of treatment. The skin was reddened and slightly swollen. As the volunteers stated that the skin area did not hurt and they wished to conclude the study, no participants were excluded at this point. However, it became clear that TEWL, skin surface pH, Epsilon^®^ and Corneometer^®^ measurements could not be performed adequately on this day due to an increasing skin reaction including exudation (Supplementary Materials Figure S3).

#### Effect of the Surfactants on Physiological Skin Parameters

**Figure 1:**
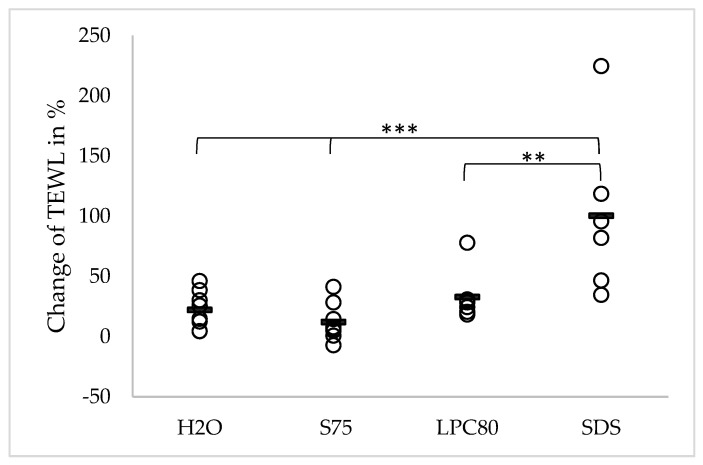
Effect of water and the three different surfactants lecithin S75, lecithin LPC80 and SDS on transepidermal water loss (TEWL) after four days of repeated exposure. Parameter changes were calculated using Equation (1) and are expressed as single values (*n* = 8, SDS values *n* = 6) and the corresponding mean. Statistically significant differences are marked with asterisks (** *p* < 0.01, *** *p* < 0.001) and were tested with a one-way ANOVA + post-hoc Tukey test with *p* < 0.05 as minimum level of significance.

The effect of repeated skin treatment with the three different surfactants and water on TEWL values is presented in Figure 1. The initial mean TEWL of all participants was 10.14 (±1.93) g/m^2^/h. After four days of treatment, the TEWL at the water-treated skin site increased slightly to 12.25 (±2.02), equivalent to a change of +22.09% (*p* > 0.05). Treatment with the two lecithins S75 and LPC80 led to mean TEWL values of 11.27 (±2.05) and 13.31 (±2.19) g/m^2^/h, equivalent to changes of +12.03% and +32.75% (*p* > 0.05). These changes did not reach statistical significance. Treatment with SDS led to a significantly increased mean TEWL of 20.41 (±6.63) g/m^2^/h, equivalent to a change of +100.31% (*p* ≤ 0.001). The increase in TEWL caused by SDS was significantly higher than the ones caused by LPC80 (*p* ≤ 0.01) and S75 or water as control (*p* ≤ 0.001).

**Figure 2:**
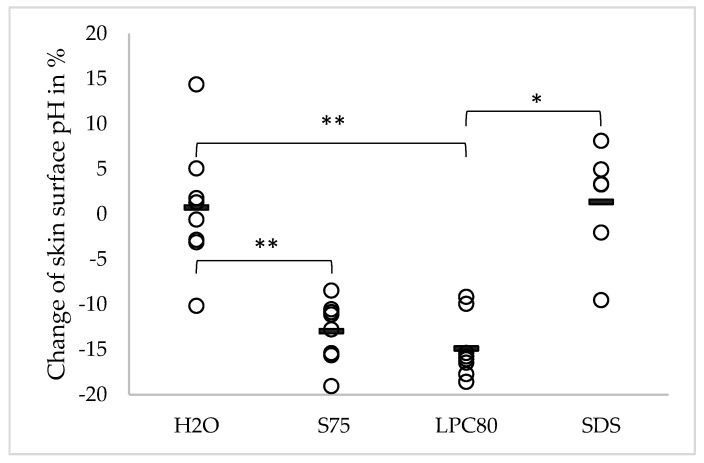
Effect of water and the three different surfactants lecithin S75, lecithin LPC80 and SDS on skin surface pH after four days of repeated exposure. Parameter changes were calculated using Equation (1) and are expressed as single values (*n* = 8, SDS values *n* = 6) and the corresponding mean. Statistically significant differences are marked with asterisks (* *p* < 0.05, ** *p* < 0.01) and were tested with a one-way ANOVA + post-hoc Tukey test with *p* < 0.05 as minimum level of significance.

The effect of the different surfactants and water on skin surface pH is shown in Figure 2. Changes in skin surface pH observed after four days of treatment are shown in percentages. The mean initial pH of all volunteers before treatment was 5.29 (±0.42). The pH did not change significantly after repeated exposure to water (5.31 ± 0.42, corresponding to an increase of +0.73%) or SDS (5.19 ± 0.18, corresponding to an increase of +1.36%). In contrast, skin sites treated with lecithins S75 and LPC80 exhibited significantly lower pH values after four days of treatment (S75: 4.59 ± 0.36, equivalent to a decrease of −12.97%; LPC80: 4.49 ± 0.36, equivalent to a decrease of −14.88%, both *p* ≤ 0.01). These differences were also statistically significant when compared to the effects of water (*p* ≤ 0.01), and in the case of LPC80 also when compared to SDS (*p* ≤ 0.05).

**Figure 3:**
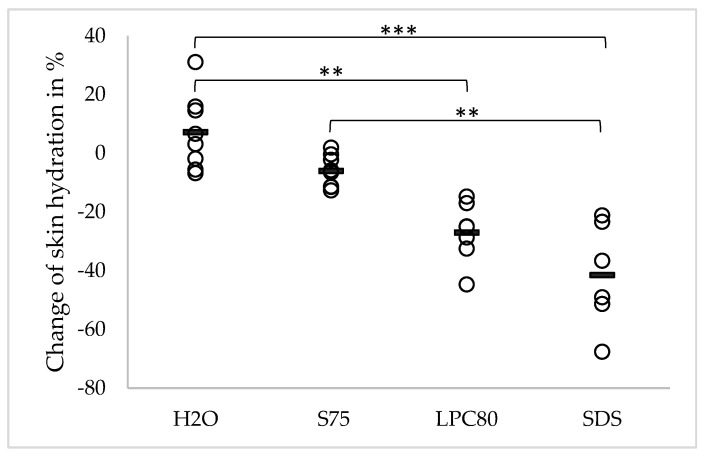
Effect of water and the three different surfactants lecithin S75, lecithin LPC80 and SDS on skin hydration after four days of repeated exposure. Parameter changes were calculated using Equation (1) and are expressed as single values (*n* = 8, SDS values *n* = 6) and the corresponding mean. Statistically significant differences are marked with asterisks (** *p* < 0.01, *** *p* < 0.001) and were tested with a one-way ANOVA + post-hoc Tukey test with *p* < 0.05 as minimum level of significance.

Changes in skin hydration caused by the repeated exposure to the surfactants and water are given in Figure 3. The observed changes are depicted in percentages. The mean skin hydration before treatment was 33.48 a.u. (±6.38). After four days of treatment, no significant changes were observed in case of water and S75. Observed values were 35.68 a.u. (±7.24) and 31.30 a.u. (±5.43), corresponding to an increase of +7.12% in case of water and a decrease of −6.08% in case of S75. In contrast, repeated exposure to LPC80 and SDS led to significantly decreased skin hydration (24.17 ± 4.28 a.u. (*p* ≤ 0.05) for LPC80 and 19.28 ± 4.79 a.u. (*p* ≤ 0.001) for SDS, equivalent to changes of −27.04% and −41.53%, respectively). Changes caused by SDS were also significantly higher than the change induced by water and S75 (*p* ≤ 0.001 and *p* ≤ 0.01). Changes in skin hydration caused by LPC80 were significantly higher than the change caused by water (*p* ≤ 0.01).

**Figure 4:**
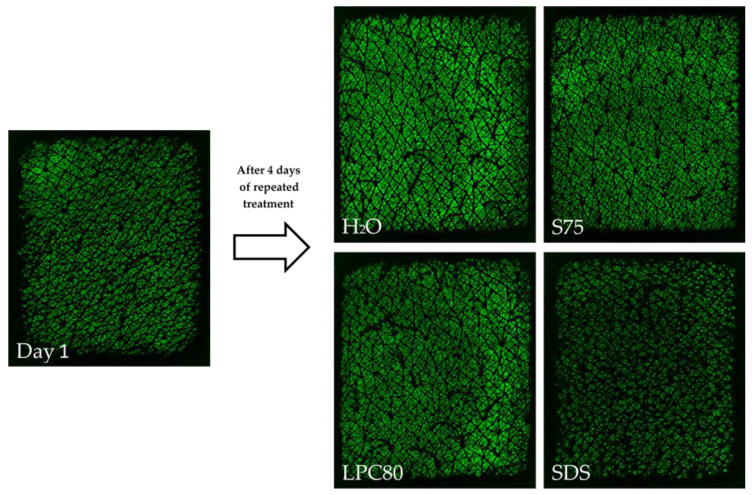
Example of capacitive images of human forearm skin acquired with the fingerprint sensor Epsilon^®^. The image on the left shows a representative measurement of untreated skin at the beginning of the study on day one; on the right-hand side, images of the volunteer’s treated skin sites after repeated treatment with water, S75, LPC80 or SDS after four days.

Skin surface hydration was also visualized using the Epsilon^®^ sensor. The results reflected the trends observed with the corneometer. However, the differences in numerical values were not as distinctive as observed with the conventional corneometer. Representative pictures of untreated and treated skin sites are shown in Figure 4. The initial mean ε value was 5.07 ± 1.70 a.u. After treatment with water and S75, the mean values increased to 7.16 ± 1.26 a.u. (*p* < 0.05) and 6.12 ± 1.69 a.u., whereas after repeated treatment with LPC80 and SDS, the mean ε values dropped to 4.15 ± 1.29 a.u. and 4.80 ± 1.25 a.u., respectively (*p* > 0.05).

The effect of treatment with the three surfactants and water on NMF and urea levels as well as water mass in percentages was determined by CRS. Focus of analysis was laid on the first 10 µm of the SC. The calculated changes in parameters of interest (NMF, urea, water) within this area were averaged and compared using Equation (1).

**Figure 5:**
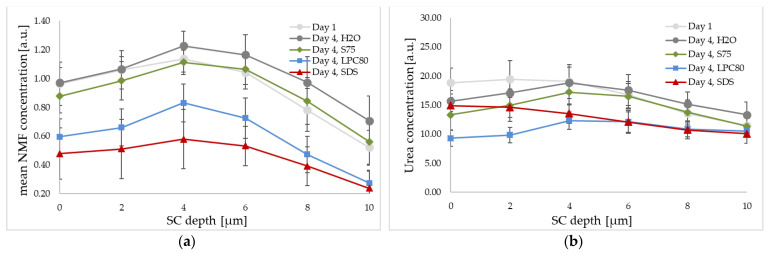
Effect of S75, LPC80, SDS and water on the relative natural moisturizing factor (NMF) (**a**) and urea concentration (**b**) of human forearm skin in vivo as determined by CRS. The NMF and urea signal intensity is given at different SC depths before the treatment (day one, light grey), and after four days of repeated exposure (day four, H_2_O in dark grey, S75 in green, LPC80 in blue and SDS in red). Values are means of *n* = 8 ± SD.

Mean changes in relative NMF concentration for the uppermost 10 µm of the SC are given in Figure 5a. An increase in NMF levels of +18.11% (±12.89) was observed after treatment with water. In the case of S75, a smaller increase of +4.48% (±18.82) was observed. In contrast, LPC80 and SDS led decreased NMF values with changes of −35.51% (±9.56) for LPC80 and −53.14% (±16.84) for SDS. These decreased values were significantly lower than the values of skin sites treated with water (control) or S75 (both *p* ≤ 0.001). 

The relative urea concentrations for the uppermost 10 µm of the SC are given in Figure 5b. The observed trends were similar as those observed for NMF values, but not as pronounced. An increase in urea levels of +4.82% was observed after treatment with water as control, while decreased values were observed after treatment with S75, LPC80 and SDS (−8.50%, −29.30% and −17.96%). The effect of LPC80 reached statistical significance when compared to the control (*p* ≤ 0. 05).

**Figure 6:**
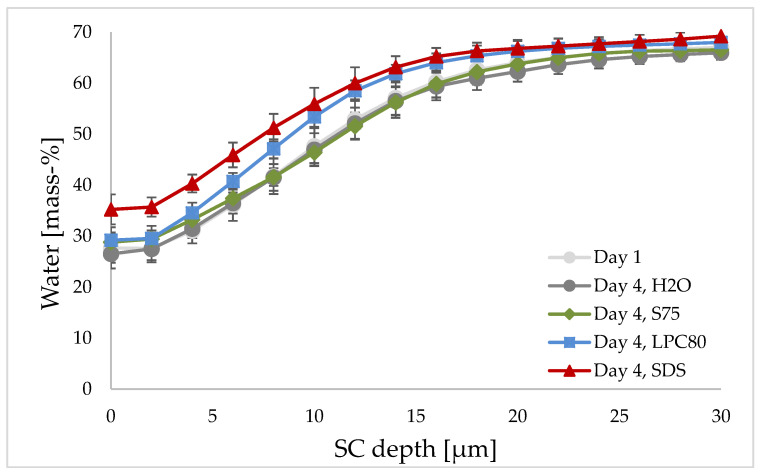
Effect of S75, LPC80, SDS and water as control on water concentration of human forearm skin in vivo as determined by CRS. Water concentration in mass-% is given at different SC depths before the treatment (day one, light grey) and after four days of repeated exposure (day four, H_2_O in dark grey, S75 in green, LPC80 in blue and SDS in red). Values are means of *n* = 8 ± SD.

The mean water concentration curves observed by CRS are given in Figure 6. Exposure to S75, LPC80 and water as control led to a water content of about 30% at the skin surface and a steady increase with increasing measurement depth to an almost constant value of 60–70% in deeper layers of the skin. However, the water increase of skin treated with LPC80 was steeper. The water content curve of SDS-treated skin started at around 35%, but increased to a similar value of around 60–70%. The water concentration within the uppermost 10 µm was significantly higher in SDS-treated skin than in water- or S75-treated skin (*p* ≤ 0.01 and *p* ≤ 0. 05).

### 3.2. Effect of Surfactants on Corneocyte Surface Morphology

**Figure 7:**
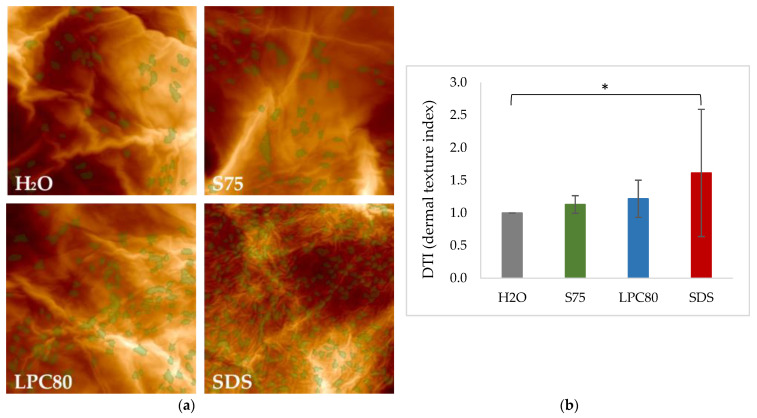
(**a**). Representative images of corneocyte surface morphology as assessed by AFM after exposure to S75, LPC80, SDS and water as control (H_2_O). Images were taken on corneocytes removed by adhesive tapes. Circular Nano Objects (CNOs) are depicted in green. (**b**) Dermal Texture Index (DTI) is defined as the average count of CNOs in a field of observation (20 μm)^2^. Values are means of *n* = 8 ± SD. Statistically significant differences are marked with asterisks (* *p* < 0.05) and were tested with a one-way ANOVA + post-hoc Tukey test with *p* < 0.05 as minimum level of significance.

AFM images of corneocyte surface morphology after four days of repeated treatment with the three surfactants and water as control were obtained. Representative examples of the visual analysis are given in Figure 7a. Treatment with water served as control. Treatment with lecithin-based surfactants S75 and LPC80 led to the formation of a higher number of CNOs when compared to the control, but the differences in regard to the calculated DTI values did not reach statistical significance (Figure 7b, *p* > 0.05). In case of treatment with SDS, the enhanced formation of CNOs was more pronounced, which can be seen form the higher number of CNOs marked in green in the corresponding images. A statistically significant increase in DTI value (*p* ≤ 0.05) was observed (Figure 7b). 

## 4. Discussion

The effects of surfactants on human skin physiology are highly relevant for our daily lives. Especially individuals with atopic predisposition, manifest pathological skin states or increased sensitivity to aggressive chemicals due to limited barrier function—e.g., in case of babies or infants—are at increased risk for skin damage caused by repeated application of surfactants. This is especially relevant when considering the current hygiene regimes introduced to counteract the COVID-19 pandemic and limit spreading of the virus via smear infection [46].

Thus, the aim of the present study was to provide new detailed knowledge on the effects of pure surfactant dispersions and to compare the impact of the aggressive anionic surfactant SDS to mild lecithin-based surfactant mixtures. Of those, one blend with reported higher tendency for skin irritation due to a higher content of monoacyl-phosphatidylcholine (S LPC80) was included for comparison with a mainly phosphatidylcholine-based surfactant mixture (S75) [47]. 

An in vivo study on healthy human volunteers was conducted over four days, with daily application of two treatments of surfactant dispersions and water as control. The results confirmed the more aggressive nature of SDS in comparison to the lecithin-based surfactants. This held true for most of the determined experimental parameters.

On two volunteers, a strong reaction with reddening of the skin and beginning exudation was observed after the fourth day of treatment. Had the reaction begun sooner, exclusion would have been the logical consequence. Due to the late occurrence of the skin reaction, the study was concluded and final measurements were performed. As the participants felt no pain and wanted to conclude the study, experiments were performed, but results from SDS-treated skin sites had to be excluded from further analysis for these two subjects due to increased surface humidity caused by inflammation and exudation. Inflammation of skin tissue and superficial humidity could affect and bias biophysical measurements such as pH, hydration or TEWL measurements. No predisposition for inflammatory or allergic skin reactions had been reported by the two individuals. This underlines the aggressive nature of SDS as a surfactant for daily use and confirms the validity of its replacement in cosmetic products for everyday use. 

Initially determined TEWL values around 10 g/m^2^/h corresponded well with previous experience and literature [39,48]. TEWL remained largely constant after treatment with the lecithins S75 and S LPC80 as well as after water treatment as control. The highest values of these three treatments were observed in case of LPS80, though they are still not statistically significant. This increase of TEWL after application of LPC80-based formulations is consistent with previous findings [47]. In contrast, treatment with SDS had a strong negative impact and led to an increase of +100%, i.e., TEWL values were twice as high after treatment with SDS over four days with values around 20 g/m^2^/h, indicating a massive deterioration of barrier function and increased loss of water [40]. This is in agreement with other in vivo observations on the effect of SDS on skin [49]. 

In regard to skin pH, representative initial values of around 5.29 were observed. Interestingly, treatment with SDS and the control water did not affect pH. In contrast, treatment with lecithin S75 and LPC80 led to significantly lower pH values after four days of treatment, with absolute values 10% to 15% lower than the initial ones around 4.59 (S75) and 4.49 (LPC80). All of these values are within physiological range and in good accordance with former in vivo studies with more complex lecithin formulations [41,47]. These findings even indicate a beneficial effect of lecithin treatment on skin health, as an acidic pH between 4 and 5 is the physiologically intended state of the skin surface pH and should be maintained. An acidic environment from 4 to 6 has been shown to deter growth of pathogenic bacteria and is essential for the function of various endogenous enzymes [50]. In contrast, the treatment with water and SDS showed a tendency towards elevated pH, which is undesired in terms of maintaining the skin microbiome and enzymatic activity at an optimum. However, it is surprising that SDS apparently had no significant impact on skin pH in this study, potentially due to the skin’s rapid potential for re-stabilization of the natural pH in case of healthy individuals.

Another parameter of interest is skin hydration within the SC. The proper barrier function of the SC and its enzymatic activity are hugely dependent on the skin’s hydration level [51]. Hydration levels influence not only skin plasticity, but also the functionality of several proteases responsible for desquamation, lipid synthesis and inflammatory responses [9,10,34]. Skin hydration as determined by corneometry was not affected after four days of treatment in case of lecithin S75 and water as control. However, significant dehydration of the SC was caused by SDS and S LPC80, where values were decreased by roughly –30% and even −40% for SDS. The same trends were obtained with the permittivity-based Epsilon^®^ sensor. Slightly higher values were observed after treatment with S75 and water while slightly lower values were obtained in case of S LPC80 and SDS. However, no clear differentiation was possible with this technique. Consistent trends, however, were observed and an inverse relationship between TEWL and skin capacitance was found [52]. 

Additionally, NMF, urea and water content of the SC was assessed using CRS. With this method, it is possible to assess certain skin properties in vivo. The confocal technique focuses the laser beam onto a specific spot below the skin surface and Raman scattered light is collected from the focal plane and analyzed, allowing the analysis of concentration and distribution of skin constituents on a molecular level [30,44,53]. Analysis of applied substances was performed considering the outermost 10 µm of the skin, as crucial changes are assumed to be reflected within this area [44]. 

The obtained spectroscopic data showed changes in NMF and urea levels throughout the first 10 µm of the SC caused by the treatments. Slightly elevated relative NMF levels were observed in case of water (+18%) and S75 (+4%). When compared to the control, LPC80 and SDS led so significantly decreased NMF values after four days of treatment (−35% and −53%). Similar trends were observed for urea levels; however, only in the case of S LPC80 a statistically significant decrease in urea levels was observed. 

NMF is a complex mixture of amino acids, their derivatives and salts. Due to its water-binding ability, it forms a natural protection against skin dehydration. Low NMF is consistent with dry skin and skin disorders such as atopic dermatitis or psoriasis [33,54,55]. The characteristic NMF concentration profile starts at higher concentrations in the outer skin zones, which decrease and remain constant from around 15 to 32 µm [56,57]. All the measured NMF profiles had this same trend; however, the SDS and LPC80 profiles started at a lower NMF concentration, leading to overall lower NMF levels. These findings are consistent with the literature [33,47,58]. These significant drops in NMF levels could be due to the denaturation of proteins of the cornified envelope, which may lead to leakage of NMF components [59,60]. 

Urea, an important component of the NMF, is essential for the preservation of skin hydration. It can amplify water absorption from the dermis to the epidermis and at the same time urea absorbs water from the external environment. Low urea levels can be found in patients with various skin diseases and are equivalent to a vulnerable epidermal barrier. Hence, the supplementation of urea through ointments and creams is widely used in dermatology [61]. Within this study, repeated exposure to S75, LPC80 and SDS led to decreased urea contents when compared to the exposure to water. Surprisingly, this decrease was only statistically significant in case of LPC80 (mean decrease of −29.30%), but not for SDS (−17.96%). The decrease in urea concentration was also found in another in vivo study by our research group using more complex formulations based on LPC80 [47]. The reasons for this specific strong effect of LPC80 remain to be clarified. In summary, it is evident that the structural differences to S75 are relevant when regarding the surfactants’ overall effect on skin. Monoacyl-phosphatidylcholine is a more potent emulsifier than phosphatidylcholine. Thus, higher irritancy can be expected when used on skin. 

The water content of the SC is largely dependent on NMF levels within the corneocytes, but also on lipid organization and corneocyte size. These factors affect TEWL, and TEWL in turn affects SC hydration. It is known that SDS affects NMF levels, disrupts lipid organization and results in higher TEWL and decreased barrier function [10,62]. Thus far, we, indeed, observed that SDS increased TEWL and lowered NMF levels. The same observation was made for monoacyl-phosphatidylcholine, but to a much lower extent.

The SC water content can also be measured using the CRS technique by calculating the SC water content from the water to protein ratio [30,63]. The water content observed by CRS for the uppermost 10 µm showed average values of 30–35% at the skin surface and a steady increase towards deeper layers. In the case of treatment with water and S75, CRS water concentration curves started at about 30% at the skin surface and increased steadily with increasing depth to 60–70%, which then stayed constant within the measured area. This correlates with the typical distribution of water in intact skin [64]. However, repeated treatment with LPC80 and SDS seemed to alter the concentration curve. With LPC80, the concentration at the skin surface was the same, around 30%; however, the gradient was steeper when compared to skin treated with water or S75. This suggests that deeper layers of the SC seemed to be more affected by LPC80. In skin treated with SDS, the water concentration curve started at around 35%, gradually rising to 60–70% in deeper skin levels. These results suggest a higher water concentration for SDS-treated skin within the first 10 µm under the skin surface. Skin sites treated with SDS tended to be reddened, with the two subjects with stronger reactions also slightly swollen potentially due to inflammatory reactions [65].

It is known that SDS interacts with SC proteins and lipids, leading to a transient swelling of corneocytes and hyper-hydration. Due to swelling, penetration of SDS as well as other ingredients is promoted into deeper layers of the skin, leading to irritation and itch [2,9]. Furthermore, SDS binds to skin proteins that are ordinarily responsible for binding and holding water, resulting in a decrease in skin hydration [9,34]. The Raman data suggested a swelling effect caused by SDS, which is in line with the observed skin reaction. This is in contrast to the permittivity-based data (Corneometer^®^, Epsilon^®^ sensor) described above, which showed dehydration. This discrepancy is most likely related to the experimental setup and the measurement duration. In case of CRS, the skin site is subjected to several minutes of occlusion during the measurement, which is not a problem with healthy skin. However, in case of an inflammatory reaction, the evaporated water in case of SDS-treated skin sites might have led to artifactually high levels of surface water.

Finally, AFM images showed impressively that all three surfactants caused changes in corneocyte morphology. The formation of CNOs was induced and DTI values were calculated. A statistically significant increase in surface protrusions, i.e., CNOs, and a resulting higher DTI was only observed in case of SDS. The increase of DTI caused by SDS is consistent with literature [34,37,38]. Additionally, DTI has recently been shown to be a reliable biomarker for irritated skin and to have a negative correlation with NMF levels, which is coherent with our findings [59]. 

To summarize, SDS had a negative impact on several skin parameters: TEWL, skin hydration/permittivity, NMF and corneocyte surface morphology. The two lecithin mixtures showed differences in regard to their effect on skin. LPC80 had a much stronger negative impact on strong physiology than S75, as seen on hydration, NMF and urea level data. This can be explained by their composition. While S75 contains a phosphatidylcholine content of approximately 70%, LPC80 is composed of 80% monoacyl-phosphatidylcholine, i.e., lysophosphatidylcholine. In phosphatidylcholine molecules, the glycerol part is esterified in two positions with different fatty acids. On a moleclar level, its critical packing parameter rather promotes the formation of lamellar layers. Monoacyl-phosphatidylcholine lacks one of the above ester bonds; it is produced through partial hydrolysis of phosphatidylcholine (Supplementary Figure S1). The glycerol part is only esterified with one fatty acid. Thus, the molecule is more hydrophilic than phosphatidylcholine, possesses a higher aqueous solubility and a packing parameter that promotes the formation of oil in water micelles. Thus, it exhibits greater emulsifying power to stabilize oil in water emulsions or solubilize lipophilic substances in general [28]. This is critical when considering its effect on skin and its potential to solubilize and extract skin lipids from the SC, thus impairing barrier function and leading to increased TEWL. Altogether, lecithin S75 seemed to be the most tolerable of the three examined surfactants, causing no measurable disadvantages. 

These results underline the importance of using skin-friendly surfactants in everyday products such as cosmetic creams. This is especially important in case of skin conditions with impaired barrier function, such as atopic dermatitis and psoriasis, which affect a considerable amount of the population. 

## 5. Conclusions

In conclusion, our results emphasize the skin compatibility of lecithins for the application in skin care products and renders them useful compounds for daily products such as shampoos, shower gels or washing detergents. Their impact on skin barrier function, especially in case of conventional phosphatidylcholine, is considerably lower than that of strong anionic surfactants or monoacyl-phosphatidylcholine. Thus, their use on sensitive skin with pre-existing conditions can be considered and should be explored further. 

## Data Availability

Not Applicable.

## Supplementary Materials

The following are available online at https://www.mdpi.com/1999-4923/13/4/436/s1. Figure S1: Hydrolysis from phosphatidylcholine to monoacyl-phosphatidylcholine. Figure S2: Structure and order of events of the study. Figure S3: Skin reaction of participant S005 to the different test substances.
