# Supplementary material for: Changes in Skin Barrier Function after Repeated Exposition to Phospholipid-Based Surfactants and Sodium Dodecyl Sulfate In Vivo and Corneocyte Surface Analysis by Atomic Force Microscopy
Source: Pharmaceutics. 2021 Mar 24;13(4):436. doi: 10.3390/pharmaceutics13040436 (PMC8063842; doi:10.3390/pharmaceutics13040436)
Supplement: Supplementary file 1 [file pharmaceutics-13-00436-s001.pdf]

pharmaceutics

Supplementary Materials: Changes in Skin Barrier Function after Repeated Exposition to Phospholipid-Based Surfactants and Sodium Dodecyl Sulfate In Vivo and Corneocyte Surface Analysis by Atomic Force Microscopy

Claudia Vater, Alexandra Apanovic, Christoph Riethmüller, Brigitte Litschauer, Michael Wolzt, Claudia Valenta and Victoria Klang

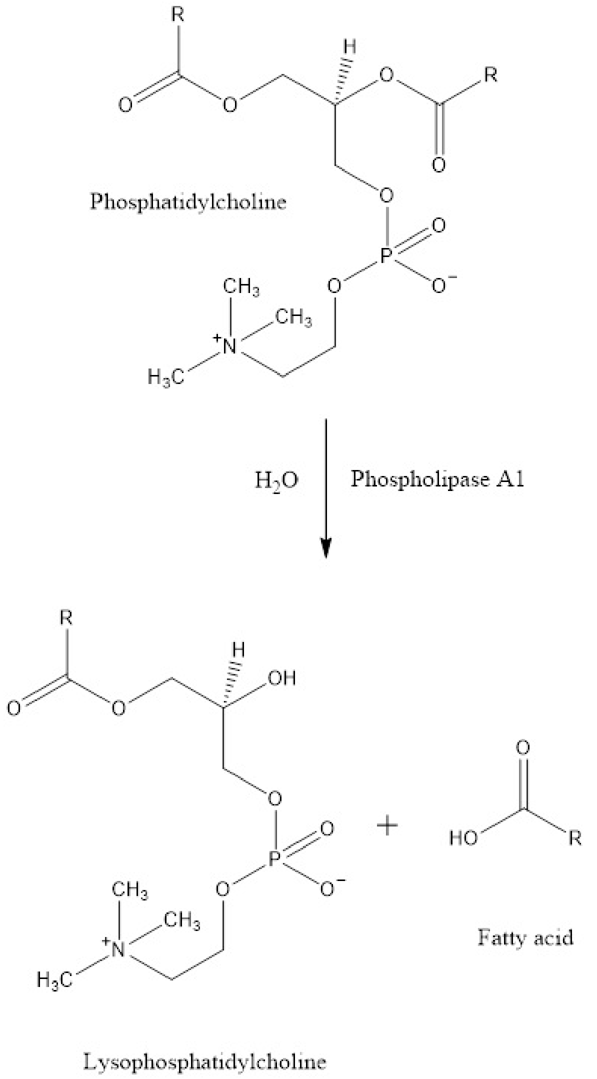

Figure S1. Hydrolysis from phosphatidylcholine to monoacyl-phosphatidylcholine.

Publisher's Note: MDPI stays neutral with regard to jurisdictional claims in published maps and institutional affiliations.

Copyright: © 2020 by the authors. Submitted for possible open access publication under the terms and conditions of the Creative Commons Attribution (CC BY) license (http://creativecommons.org/licenses/by/4.0/).

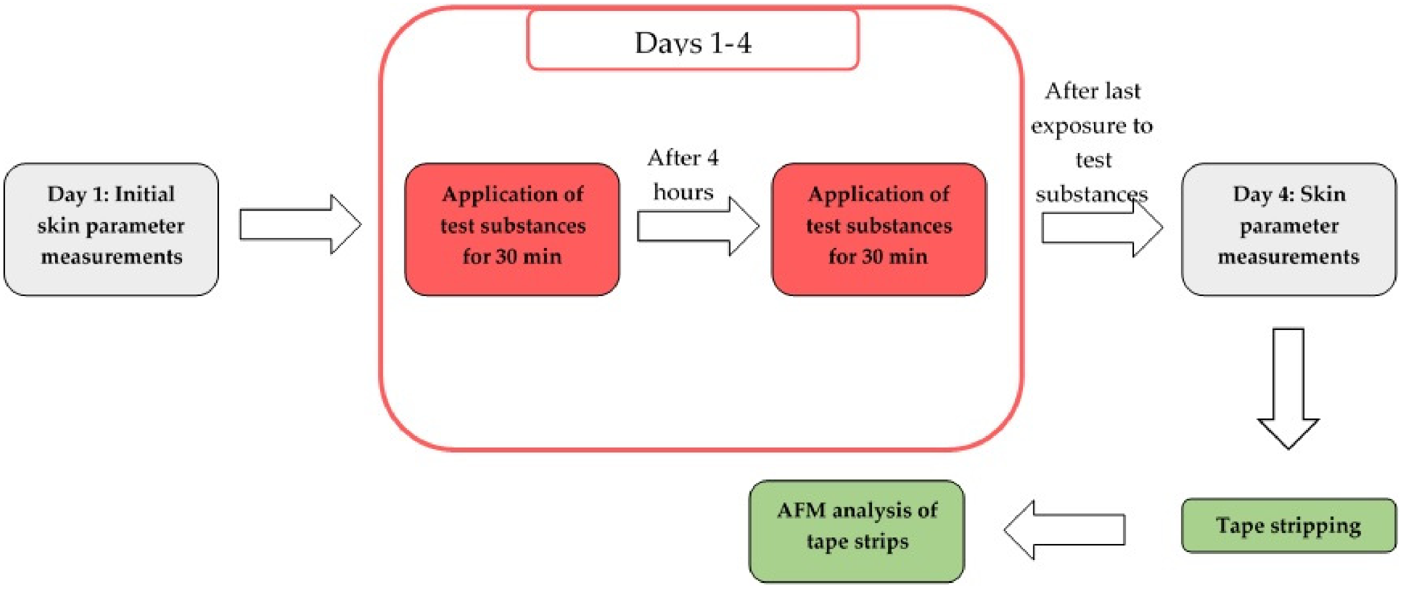

Figure S2. Structure and order of events of the study.

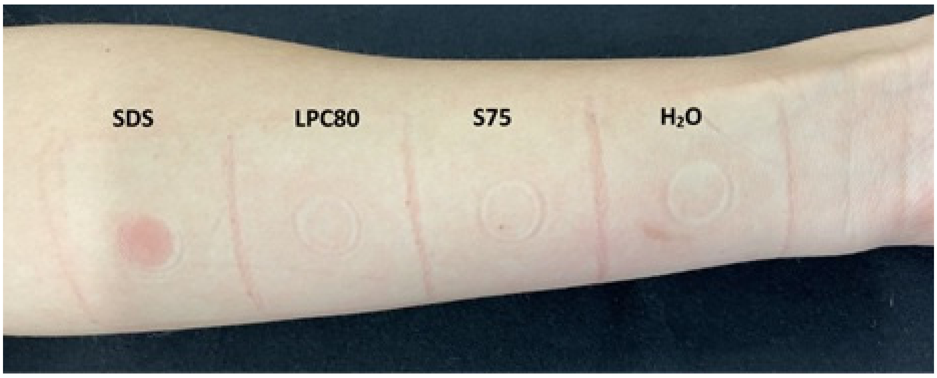

Figure S3. Skin reaction of participant S005 to the different test substances.
